# Data on screening and identification of genetically modified papaya in food supplements

**DOI:** 10.1016/j.dib.2016.08.028

**Published:** 2016-08-20

**Authors:** Theo W. Prins, Ingrid M.J. Scholtens, Arno W. Bak, Jeroen P. van Dijk, Marleen M. Voorhuijzen, Emile J. Laurensse, Esther J. Kok

**Affiliations:** a RIKILT Wageningen UR, Akkermaalsbos 2, 6708 WB Wageningen, Netherlands; b Netherlands Food and Consumer Product Safety Authority (NVWA), Akkermaalsbos 4, 6708 WB Wageningen, Netherlands; c Netherlands Food and Consumer Product Safety Authority (NVWA), Catharijnesingel 59, 3511GG Utrecht, Netherlands

## Abstract

This article contains data related to the research article entitled “A case study to determine the geographical origin of unknown GM papaya in routine food sample analysis, followed by identification of papaya events 16-0-1 and 18-2-4” (Prins et al., 2016) [1]. Quantitative real-time PCR (qPCR) with targets that are putatively present in genetically modified (GM) papaya was used as a first screening to narrow down the vast array of candidates. The combination of elements P-nos and nptII was further confirmed by amplification and subsequent sequencing of the P-nos/nptII construct. Next, presence of the candidate GM papayas 16-0-1 and 18-2-4 were investigated by amplification and sequencing of event-spanning regions on the left and right border. This data article reports the Cq values for GM elements, the nucleotide sequence of the P-nos/nptII construct and the presence of GM papaya events 18-2-4 and/or 16-0-1 in five samples that were randomly sampled to be analysed in the framework of the official Dutch GMO monitoring program for food.

**Specifications Table**

**Table:** 

Subject area	Biology
More specific subject area	Biotechnology, qPCR
Type of data	Table, Figure
How data was acquired	Data are Cq qPCR values calculated with Bio-Rad CFX software, and the nucleotide sequence of P-nos/nptII amplicon generated by Sanger sequencing
Data format	Analysed
Experimental factors	DNA isolated from 5 papaya food supplements
Experimental features	qPCR and Sanger sequencing
Data source location	Wageningen, Netherlands
Data accessibility	Data is within this article and available at GenBank via the accession numbers GenBank: KU376437, GenBank: KU376438, GenBank: KU376439, GenBank: KU376440, GenBank: KU376441, GenBank: KU376442

**Value of the data**

• Data presented here provide a screening strategy for the identification of unknown genetically modified papaya.
• We show Cq (quantification cycle) values of common elements putatively present in genetically modified papaya to show that a combination of qPCRs can narrow down the suspect known GM papaya events.
• Using the forward primer of a promoter and a reverse primer of a coding sequence in PCR and subsequent sequencing of the amplicon reveals part of the construct P-nos/nptII.
• We show that PCR with event-specific primers for the left and right border of GM papaya 16-0-1 and 18-2-4 confirms the presence or absence of the GM papaya(s) in food samples.

## 1 Data

**Fig. 1:**
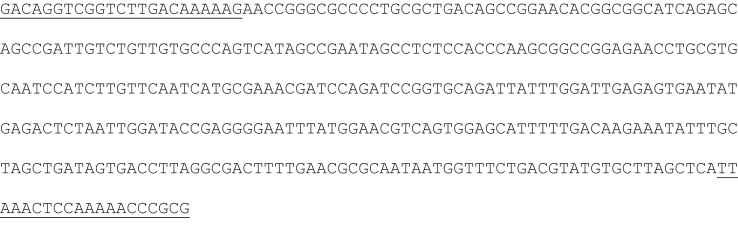
P-nos/nptII 368 bp amplicon generated by conventional PCR combining the nptII forward primer and the P-nos reverse primer (underlined).

The data presented in this article show the Cq values of the available qPCRs for known GM elements in GM papaya (Table 1). The nucleotide sequence (partial) of the P-nos/nptII construct (Fig. 1), and the event-specific borders of GM papaya are presented (Table 2). Data were obtained by extracting DNA from five papaya samples.

## 2 Experimental design, materials and methods

### 2.1 Materials

The analytes consisted of five different papaya food supplements that were sold as capsules with dried and powdered papaya, or papaya powder. See [1] for detailed information.

### 2.2 DNA extraction

According to [1], in brief, the Promega Wizard^®^ Magnetic DNA Purification system for food (Promega, Madison WI, USA) was used according to the manufacturer׳s protocol with minor modifications. A Nanodrop spectrophotometer (ND-1000, Montchanin, DE, USA) was used to quantify and assess the purity of the DNA.

### 2.3 Primers and probes

Primer and probes used for the detection of targets described in Table 1, the amplification and sequencing of the partial P-nos/nptII cassette (Fig. 1), and the amplification and sequencing of the left border and right border of events 16-0-1 and 18-2-4 (Table 2), are given in Prins et al. [1].

### 2.4 PCR reactions

qPCR was performed on a CFX (Bio-Rad). qPCR trace files were analysed with the Bio-Rad CFX manager software version 3.0. For full PCR conditions, consult [1].

### 2.5 Amplicon analysis

Samples were bidirectionally sequenced by Macrogen Europe (Amsterdam, the Netherlands) using the forward and reverse primers. Sequence data were analysed using Staden Package software (version 1.6.0: for trimming, aligning, and consensus building), BLAST [2] and Clustal Omega [3].

## Figures and Tables

**Table 1:** Quantification cycles (Cq) for five papaya samples (5 ng DNA/PCR) with different TaqMan qPCR methods.

**Sample**	**Method**								
	**P-35S**	**T-35S**	**P-nos**	**T-nos**	**nptII**	**CHY**	**55-1 /63-1 construct**^a^	**Event 55-1**^a^	**Huanong No.1**^b^
A	29.78	–	31.85	31.84	32.55	27.43	–	–	–
B	32.57	–	33.86	34.94	34.28	28.38	–	–	–
C	29.47	–	32.22	30.79	31.09	25.70	–	–	–
D	33.92	–	35.36	35.43	37.16	26.15	–	–	–
E	29.46	–	31.70	30.79	31.03	27.24	–	–	–

−: not detected.

a The 55-1 /63-1 construct- and 55-1 event-specific methods were positive with 55-1 reference material while wild type papaya and water were negative.

b No positive Huanong No.1 reference material was available. The Huanong No.1 event-specific method was negative with wild type papaya and water.

**Table 2:** Summary of events 16-0-1 and 18-2-4 LB and RB (left border and right border) conventional PCR results on DNA of five papaya food supplement samples. Nucleotide sequences spanning the integration site were filed in GenBank as KU376439-KU376441.

**Sample**	**Method**			
	**16-0-1 LB (105** **bp)**	**16-0-1 RB (249** **bp)**	**18-2-4 LB (140** **bp)**	**18-2-4 RB (309** **bp)**
A	+	+	–	–
B	+	+	–	–
C	+	+	–	–
D	+	+	+	+
E	+	+	–	–

−: not detected; +: detected.
