# Supplementary material for: Data on screening and identification of genetically modified papaya in food supplements
Source: Data Brief. 2016 Aug 20;9:43–6. doi: 10.1016/j.dib.2016.08.028 (PMC5011157; doi:10.1016/j.dib.2016.08.028)
Supplement: Supplementary file 1 — Supplementary material [file mmc1.docx]

DIB-D-16-00555 revision 160801

'Conflicts of interest: none'.
